# Repetitive Transcranial Magnetic Stimulation for Treatment-Resistant Depression: Recent Critical Advances in Patient Care

**DOI:** 10.1007/s40501-021-00238-y

**Published:** 2021-03-11

**Authors:** Camila Cosmo, Amin Zandvakili, Nicholas J. Petrosino, Yosef A. Berlow, Noah S. Philip

**Affiliations:** 1 VA RR&D Center for Neurorestoration and Neurotechnology, Providence VA Healthcare System, 830 Chalkstone Ave, Providence, 02908 USA; 2 Department of Psychiatry and Human Behavior, Alpert Medical School of Brown University, Providence, RI USA

**Keywords:** Major depressive disorder, Depression, Transcranial magnetic stimulation, Theta burst stimulation

## Abstract

**Purpose:**

Transcranial magnetic stimulation (TMS) is an evidence-based treatment for pharmacoresistant major depressive disorder (MDD). In the last decade, the field has seen significant advances in the understanding and use of this new technology. This review aims to describe the large, randomized controlled studies leading to the modern use of rTMS for MDD. It also includes a special section briefly discussing the use of these technologies during the COVID-19 pandemic.

**Recent findings:**

Several new approaches and technologies are emerging in this field, including novel approaches to reduce treatment time and potentially yield new approaches to optimize and maximize clinical outcomes. Of these, theta burst TMS now has evidence indicating it is non-inferior to standard TMS and provides significant advantages in administration. Recent studies also indicate that neuroimaging and related approaches may be able to improve TMS targeting methods and potentially identify those patients most likely to respond to stimulation.

**Summary:**

While new data is promising, significant research remains to be done to individualize and optimize TMS procedures. Emerging new approaches, such as accelerated TMS and advanced targeting methods, require additional replication and demonstration of real-world clinical utility. Cautious administration of TMS during the pandemic is possible with careful attention to safety procedures.

## Introduction

Neuropsychiatric disorders continue to be the third leading cause of disability worldwide, with 10.4% of total global burden, measured by global disability-adjusted life years (DALYs) [1]. Of these disorders, major depressive disorder (MDD) is associated with the greatest burden, corresponding to 2.54% of global DALYs and 3.7% of all US DALYs [2, 3]. An estimated 264 million people are stricken by depressive disorders worldwide [4]. In US adults, the lifetime prevalence of MDD was found to be 20.6%, with most individuals presenting moderate or severe courses and substantial impairment [5]. These data indicate the seriousness of this mental health disorder and demonstrate the importance of developing novel and effective therapeutic approaches.

Treatment-resistant depression (TRD), or more specifically pharmacoresistant MDD, is defined as the lack of remission despite multiple (generally defined as >2) trials of a tolerable and evidence-based treatment, and is associated with significant disability. As a classic exemplar study of TRD, the Sequenced Treatment Alternatives to Relieve Depression (STAR*D) trial revealed that 63.2% did not remit following the first trial; up to a third never achieved remission [6]. Lack of effectiveness is another important issue in depression management. Effectiveness consists in ≥50% reduction in baseline symptom severity [6, 7]; compared to placebo, antidepressants have an effectiveness of up to 30%. This figure is even lower when the outcome under investigation is remission, defined as meeting threshold criteria on standard rating scales [8]. Based on these data, current TRD treatment usually features various augmentation strategies, such as using mood stabilizers and/or antipsychotics, which also results in polypharmacy, with potential interactions and associated safety concerns.

One novel approach to TRD management over the last decade is repetitive transcranial magnetic stimulation (rTMS). This was cleared by the US Food and Drug Administration (FDA) for patients with pharmacoresistant depression in 2008. rTMS is a non-invasive brain stimulation procedure that applies repeated magnetic pulses over the scalp to generate an electrical current in the cortex, provoking electrophysiological effects that modify the neural excitability in the target area and correlated brain networks [9, 10, 11••, 12]. Its safe profile (particularly lack of systemic side effects associated with pharmacotherapy), cost-effectiveness, and better focality are some of its advantages over other neuromodulation techniques, such as electroconvulsive therapy [13–17].

Early work suggested the antidepressant effects of rTMS were exclusively related to modulation of left and right dorsolateral prefrontal cortex (DLPFC) excitability [18–22], based in theory that depression resulted from hypoactivation of the left prefrontal region and increased activity in the right DLPFC [23–27, 28•]. With a progressive understanding of the pathophysiology of depression and contribution of intrinsic connectivity networks to depression [29–32], functional mapping was applied in later trials to investigate neural mechanisms underlying TMS therapeutic effects [33–35]. This work reported changes in brain regions distal to the site of stimulation, such as the thalamus and amygdala, and indicated that the therapeutic mechanism of action is related to polysynaptic (i.e., “downstream”) effects [36, 37].

Although numerous trials have now demonstrated the effectiveness of rTMS monotherapy or augmentation [20, 22, 38–41], not all findings have been robust [42–44], and important questions persist regarding optimal use. We reviewed and summarized the most recent findings of randomized rTMS clinical trials for pharmacoresistant depression, addressing the optimization of parameters, potential neurophysiological biomarkers, and ongoing areas of research such as emerging neuromodulation techniques.

## Technical aspects of TMS

rTMS is a non-invasive approach to modulate neural circuitry using electromagnetic fields. By running an alternating electrical current through a coil placed on the scalp of the patient, a focal and fluctuating magnetic field is generated which, in turn, induces an electrical current (following Faraday’s law). This induction occurs primarily in the cortical grey matter neurons of the underlying target region. When these currents are run rapidly and in succession, they comprise the magnetic “pulses” of rTMS, with each pulse achieving a peak magnetic field strength of ~1.5 Tesla [45]. The target region, most commonly the DLPFC for depression, is the area of cortex where the induced electrical field is maximal, and successful targeting depends on accurate surface placement of the TMS coil and coil geometry. One of the greatest challenges in targeting derives from a fundamental property of coil design: greater depth, achieved by larger dimensions of a coil, results in a less focal electrical field [46•]. While the figure-8 coil design, the most commonly used coil in clinical TMS, significantly improved focality, the so-called depth-focality trade-off remains an important limitation with depth of stimulation around 2–3 cm [47].

Several parameters define rTMS, including frequency, intensity, train duration, intertrain interval, and session total. Frequency refers to the number of magnetic pulses delivered over time expressed in Hertz. While high-frequency TMS at 10 Hz targeting the left DLPFC has long demonstrated efficacy [20, 48], low frequency (1 Hz) targeting the right DLPFC and bilateral TMS have also been shown to be effective [49, 50••]. Stimulation intensity is expressed as a percentage of the motor threshold, i.e., the minimal amount of energy delivered to the primary motor cortex that is required to elicit a motor response typically in the contralateral hand (called the motor threshold). A “train” is a series of pulses. Train duration refers to the amount of time in which a series of pulses are delivered. Intertrain interval refers to the time between trains. Lastly, session total is the number of pulses delivered in a single session. In clinical TMS for TRD, patients most commonly receive 10-Hz stimulation to the left DLPFC at an intensity of 120% of motor threshold with 4-s trains and 26-s intertrain intervals for 3,000 pulses. Standard sessions last 37.5 min and a treatment is 5 days per week for 4–6 weeks. These settings mimic those used in the pivotal studies described below, although recent work indicates that slightly shorter intertrain intervals are effective. [51] Still, the optimal parameters for treatment are not completely understood and other types of TMS have evidence for use in pharmacoresistant depression. For example, theta burst stimulation (TBS) delivers pulses at 50-Hz triplets repeated at 5 Hz in 2-s trains every 10 s, parameters designed to mimic the endogenous theta rhythm of hippocampal pyramidal neurons [52•]. Recently shown to be non-inferior to TMS in the treatment of depression [53], TBS is a promising development as it offers sessions that last <10 min, decreasing patient burden and improving cost-effectiveness.

## TMS studies

To date, there are seven TMS systems cleared for use in TRD: NeuroStar, BrainsWay (H1-coil), MagVenture, CloudTMS, Apollo, Nexstim, and Magstim. Since October 2008 (when TMS was first FDA cleared), over 360 studies investigating the application of TMS in depression have been published. Among those, there are more than 150 trials and 47 meta-analyses, with 29 randomized clinical trials (RCTs) and 7 meta-analyses specifically addressing individuals with TRD. The studies selected for the current comprehensive review include the state-of-the-art in the field, in addition to relevant RCTs published in the last two decades addressing the effects of TMS in the modulation of depressive symptoms.

### Pioneering works

In 1995, George et al. performed the first trial to examine the clinical application of rTMS. In this pilot study, six patients with TRD (five of whom had bipolar disorder) received at least five consecutive sessions of rTMS (80% motor threshold [MT] [54]; 800 stimuli at 20 Hz cycles), applied over the left prefrontal cortex. One patient remitted and clinical improvement was observed in another. Interestingly, the only remission was observed in the individual with unipolar depression. Given the design restrictions and its small sample, the conclusions were limited, but suggested rTMS was well-tolerated with potential antidepressant properties. This initial observation was confirmed by more robust trials [38, 39], including a large multisite double-blind, sham-controlled randomized clinical trial [20]. Notably, prior to this pilot trial, two other studies have addressed transcranial magnetic stimulation antidepressant effects, but both consisted of case reports and had applied single pulse TMS [55, 56].

O’Reardon et al. conducted a pivotal double-blind, randomized, sham-controlled, multisite study, including 301 antidepressant-free individuals with MDD who had failed at least one antidepressant [48]. The authors found that, when compared to sham, rTMS applied to the left DLPFC safe and effective for TRD. This trial, sponsored by Neuronetics (NeuroStar TMS Therapy System, Neuronetics Inc., Malvern, PA, USA), was the foundation of the initial FDA clearance for TMS use in pharmacoresistant major depression. The stimulation protocol consisted of five TMS sessions per week, repetition rate of 10 Hz, applying 120% of MT, 3000 pulses per session, for 4–6 weeks (acute treatment phase), followed by a 3-week taper period. Symptom improvement was observed at 4 weeks (17-item Hamilton Depression Rating Scale: *p*=.006; and 24-item (HAMD24): *p*=.012; Montgomery-Asberg Depression Rating Scale (MADRS): *p*=.038, with post hoc correction for baseline score imbalance) with an even more significant clinical response (HAMD17: *p*=.005; HAMD24: *p*=.015), and remission rates (outcomes for active vs. sham; MADRS: 14.2% vs. 5.2%, HAM-D17: 15.5% vs. 7.1%, HAMD24; 17.4% vs. 8.2%), at the end of 6 weeks of intervention, except for MADRS (*p* = .052)). Furthermore, rTMS was proven to be safe and well tolerated, with side effect–related dropout rate as low as 4.5%, without reports of serious adverse events such as seizure or death [48]. Results from this study were replicated by a multisite NIMH-sponsored study, which found comparable response, remission, and safety outcomes [20].

### TMS effectiveness

In the first large, naturalistic study of TMS, Carpenter et al. investigated the effectiveness of rTMS in TRD in a multisite study [57]. Three-hundred seven individuals were treated using parameters used in O’Reardon et al., applied over the left DLPFC in the majority of the patients (i.e., 208 subjects), with a few exceptions when a sequential bilateral stimulation or right-sided rTMS was chosen when nonresponse was seen following left-sided application. In addition to completing the acute phase treatment, 86.3% (265 subjects) of the original sample joined a 52-week follow-up study, also using a naturalistic approach.

During the acute course, patients had on average 28.3 (SD=10.1) rTMS sessions, consisting of 42 days (SD=14.2), resulting in significant improvement in depression symptoms and severity as shown by the (a) Clinical Global Impression-Severity scale (CGI-S)—change from baseline to endpoint (*p*<.0001), response rate 58.0%, and remission rate 37.1%; (b) Inventory of Depressive Symptomatology, Self-Report (IDS-SR)—response rate 41.5%, and remission rate 26.5%; and (c) 9-Item Patient Health Questionnaire (PHQ-9)—response rate 56.4%, and remission rate 28.7%. Only one major adverse event was documented. A sleep-deprived patient, who was on sertraline, bupropion, and dextroamphetamine/levoamphetamine, had one seizure in her 10th session, likely due to a lower seizure threshold in the setting of multiple contributors. This study confirmed what had been seen in prior large controlled trials [20, 48, 58–60], namely that rTMS is safe, is well tolerated, and is an effective treatment for TRD.

### Deep TMS

Despite multiple trials showing the effectiveness of rTMS for TRD [20, 22, 38–41], modest findings or even lack of effect has been observed [42–44]. Within this context, questions have arisen about whether more modest effects of rTMS are associated with less brain penetration (see section above describing the depth/focality trade-off) [61]. As an attempt to expand the amount of stimulated tissue, the H-coil was developed with the aim of modulating deeper brain regions, creating a new rTMS modality referred to as “deep” transcranial magnetic stimulation (dTMS) [62]. The H-coil allows stimulation of somewhat deeper cortical layers and wider brain areas, as demonstrated in mechanistic studies [63, 64]. Levkovitz et al. carried out the first trial to evaluate the safety and effectiveness of dTMS in TRD [61]. Sixty-five subjects were randomly assigned to four different arms (with all groups receiving 1,680 stimuli at 20 Hz, per session): (a) H1-coil, 120% MT, predominantly stimulating the left prefrontal cortex; (b) H2-coil, 120% MT, inducing bilateral stimulation; (c) H1L, 120% MT, applied specifically over the left PFC; and (d) H1L, 110% MT, left PFC. After tapering the antidepressants for 2 weeks, patients completed 4 weeks (20 sessions) of active dTMS, with no sham. Depression symptoms improved significantly in those assigned to dTMS at 120% MT (all *p*<.001), with more robust findings observed in those submitted to unilateral stimulation (H1-coil, response rate: 47%; remission: 42%; H1L-120%, response rate: 60%; remission: 50%), when compared to bilateral (H2-coil group, response rate: 30%; remission: 10%). Over half of participants attended a follow-up assessment at 3 months, and reported sustained improvement. No major adverse events or cognitive impairments were observed, and these findings led to FDA clearance of the H1-coil system (Brainsway Deep TMS Therapy System, Jerusalem, Israel) in January 2013. Later, the same research group conducted a large double-blind randomized sham-controlled multicenter trial that confirmed the efficacy, safety, and prolonged effects of dTMS [65].

Whether one particular coil design can produce superior results is an important and unanswered question for the field. To compare the safety and antidepressant efficacy of rTMS applied using a figure-8 coil vs. an H1-Coil, Filipčić et al. performed an industry-independent randomized controlled trial, and found no differences in remission rates between the two coils (all *p*>.1) [66]. They reported higher response rates with dTMS (*p*=.04), although both modalities yielded remission rates that are typically higher than those reported in other studies. Regardless, the comparable remission outcomes provide empiric support that clinical outcomes are more likely associated with the “downstream” or polysynaptic effects of TMS, which may be independent of the devices used.

### Theta burst stimulation

Theta burst stimulation is novel and so-called second-generation rTMS modality [67, 68]. It was initially investigated as a neurophysiologic tool as described above, with its primary differentiating factor being that it can modulate synaptic plasticity with effective results in a very short period of time (typically 3–10 min for an entire “dose,” compared to the standard 37.5 min required for an rTMS session) [68, 69]. The first human study to TBS, performed by Huang et al., included delivery of short bursts of a high-frequency (50 Hz) TMS, repeated at intervals of 200 ms (5 Hz), at 80% MT, in three different patterns (intermediate TBS (imTBS); continuous TBS (cTBS); and intermittent TBS (iTBS)) [67]. iTBS was found to yield electrophysiological changes in the motor cortex when administered as intermittent (iTBS; 2 s train, repeated at 10s) or continuous (cTBS; 40s train of uninterrupted stimulation); stimulation yielded robust long-term potentiation (iBTS) or long-term depression (cTBS)-like activity. This led to the first randomized controlled trial of TBS for depression by Li et al. [70]. They randomized sixty patients with TRD to four groups—(a) cTBS, (b) iTBS, (c) a combination of cTBS and iTBS, and (d) sham TBS, with 15 patients per group. All patients received 2 weeks of stimulation, and they found that depression improved in all groups, but those who received iTBS consistently demonstrated superior outcomes.

The largest study of TBS to date was performed by Blumberger et al., who conducted a large randomized multisite non-inferiority trial to compare iTBS versus rTMS [53]. In this trial (*n*= 404), patients received up to 30 treatments of 10 Hz rTMS (120% TMS, at 10 Hz, 3,000 pulses/session, duration: 37.5 min) or iTBS (120% MT, 50 Hz bursts, at 5 Hz, 600 pulses/session, duration: 189 s) over the DLPFC. Statistically significant response and remission rates were detected in the iTBS group (HRSD-17—reduction from baseline to endpoint: 10.1 points; response rate: 49%; and remission: 32%), as well as in the 10 Hz rTMS group (HRSD-17—reduction from baseline to endpoint: 9.9 points; response: 47%; and remission rate: 27%), confirming the study hypothesis that iTBS was non-inferior to 10 Hz rTMS in improving depressive symptoms. In regard to safety, headache was the most prevalent adverse event, with no differences in side effects and tolerability between groups [53]. This trial led to FDA clearance of iTBS for TRD.

### Laterality

Stimulation laterality is an important and understudied area in rTMS research. TRD rTMS protocols have predominantly employed three different protocols: (a) unilateral high-frequency rTMS (HF-rTMS, ≥5 Hz) targeting the left DLPFC; (b) unilateral low-frequency rTMS (LF-rTMS, 1 Hz) to the right DLPFC; and (c) bilateral, by sequentially applying HF-rTMS to the left and LF-rTMS to the right DLPFC [20, 71–74]. All these modalities appear superior to sham [20, 71–74], yet studies comparing these protocols have produced differing outcomes. Analogous results in regard to depressive symptoms improvement were observed in studies evaluating left (HF-rTMS) vs. right stimulation (LF-rTMS) [72, 73], and bilateral stimulation has generally not been shown to be superior to unilateral rTMS [27, 75, 76, 77••]. However, in a network meta-analytic approach, Brunoni et al. indicated bilateral rTMS might be more effective compared to HF-rTMS (OR= 4.02; 95% CI= 1.3–12.35) [78••]. Interpretation of this finding is mitigated by issues related to network meta-analyses, where interventions can be contrasted yet never prospectively tested against each other. Recently, evidence has emerged that supports the idea of equivalence between left HF-rTMS and right-sided LF-rTMS from Berlow et al. [79]. This is an area of important inquiry as LF-rTMS devices are considerably less expensive and could be made more portable to address patient needs during the pandemic (see COVID19 section, below).

### Durability of effects

Durability of rTMS-related antidepressant effects is also an important consideration. The long-term effects of rTMS were assessed in a year-long follow-up study [80], revealing a sustained response as shown by the clinical outcomes: (a) CGI-S—change from end of acute phase to endpoint (*p*=.0269), response rate 67.7%, and remission rate 45.1%; (b) Inventory of Depressive Symptomatology, Self-Report (IDS-SR)—response rate 44.1%, and remission rate 29.3%; and (c) 9-Item Patient Health Questionnaire (PHQ-9)—response rate 60.7%, and remission rate 37.0%, with response/remission rates at 12 months endpoint similar to acute outcomes. After completing acute rTMS, 36.2% of subjects required at least 1 additional rTMS session over the period of 1 year, with an average of 16.2 sessions (SD=21.1). Sixty-two and a half percent of 120 individuals, who responded or remitted following the acute course, remained responsive in all the assessments (at 3, 6, 9, and 12 months). No serious adverse events were observed. In addition to validating prior findings on rTMS effectiveness and safety, this study showed that rTMS yields durable effects in TRD patients and that patients can respond to retreatments. Additional studies have shown similar findings, endorsing its long-lasting benefits [81, 82].

### Emerging interventional techniques

In the past two decades, interventional psychiatry has advanced significantly, resulting in the emergence of innovative neuromodulation techniques. Groundbreaking trials have investigated the application of these techniques in several neuropsychiatric disorders with promising results. Particularly in treatment-resistant depression, novel approaches have been explored. Preliminary data has indicated the potential effectiveness of transcranial direct current stimulation (tDCS) [83–85], magnetic seizure therapy (MST) [86–89], transcranial photobiomodulation (t-PBM) with near-infrared (NIR) [90, 91], and transcranial focused ultrasound (tFUS) [92, 93], as well as of low field magnetic stimulation (LFMS) [94, 95]. Additionally, vagus nerve stimulation (VNS) has been cleared by the FDA for TRD [96–98]. As a comprehensive approach of different neuromodulation techniques for TRD is beyond the scope of this review, please see the aforementioned references for further information.

## Challenges

### Stimulation target

One important area that requires clarification is the identification and engagement of the stimulation target. The field has progressively moved from a simple, anatomically based targeting method, to coil placement based upon individual skull anatomy (Beam F3) [99]. Within the last several years, there is an increasing body of evidence indicating that functional connectivity relationships between the subgenual anterior cingulate (sgACC) and DLPFC may be able to yield an individualized stimulation target [100••, 101]. This approach holds the promise of improving clinical outcomes. Major challenges in this space will be prospectively testing whether these imaging methods can provide superior outcomes to standard of care targeting, and whether the improvements gained are sufficiently robust to outweigh the real-world cost of employing these technically advanced approaches.

### Accelerated rTMS

“Accelerated TMS” has been the subject of considerable attention. This approach includes the administration of many TMS sessions throughout the day, with the general idea to provide an entire course of TMS to a patient in a few days, as opposed to 6–8 weeks. Holtzheimer et al. performed the first study in this space, and administered 15 low-dose rTMS sessions over 2 days, and found fair response and remission rates [102]. Two crossover studies (Baeken et al. [103] and Desmyeter et al [104]) found accelerated TMS to be safe and feasible, and a recent clinic-based study compared once daily vs. twice daily TMS although found no group differences [105••]. Most recently, a small unblinded study by Cole et al. indicated that iTBS could be administered many times over a single day [106]. In this study, 19 patients received TBS (1,800 pulses per session, 50-min intersession interval, 90% MT adjusted for cortical depth) for 10 sessions daily and targeted to the DLPFC using functional neuroimaging. They reported very high remission rates (*n*=19; 86.4%), although loss of efficacy at 1 month. If replicated in a randomized controlled trial, this holds significant promise as a new TRD treatment.

### Predictors of response: biomarkers

TMS is very effective, yet costly in the real world; it required significant financial and time commitment from the patient and providers. Over the last decade, there has been significant interest in developing predictors of response. It is important to note that the vast majority of identified predictors have largely been unsuccessful in their goal. For example, patient-level predictors, such as treatment resistance, age, and sex, all have been associated with TMS response (or lack thereof) [107–109], with later data either refuting or at least indicating difficulty replicating these findings (for a comprehensive review, please see [110•, 111]). To this end, we highlight a few of the more recent advances in predictor development, with a focus on biomarkers—biological markers that can predict or characterize treatment response. We acknowledge that non-replication is also a feature of this literature (e.g., [112, 113••]) and provide the below as examples.

#### Neuroimaging

Several studies have suggested that functional neuroimaging may provide patterns of connectivity associated with TMS response (for a review, please see [114]). The most notable and consistent change associated with rTMS across studies is in the Default Mode Network [115, 116•, 117, 118]. Also notable are studies that use clustering and machine learning algorithms to identify brain-based variants of depression [119•]. This approach can lead to finding specific sub-types of depression, with network properties associated with TMS response. The most immediate use of this research has to help identify improved ways to target rTMS.

#### Electrophysiological

Recent studies have used EEG to track TMS-emergent changes in cortical networks. EEG interpretation can be complicated and unintuitive as this signal is complex, is time varying, and has low spatial resolution, and there has been little success in finding clear EEG biomarkers for depression [120•]. Machine learning and other data-driven approaches of value analyze and extract salient features of EEG data to identify predictors and mechanisms of treatment. This approach has been used in several recent studies, where EEG-based functional connectivity can identify and predict clinical outcomes [121, 122]. Although these methods show promise, further studies need to validate the outcomes and standardize the approach. EEG holds potential for clinical use, as it is more available and implementable in clinical settings.

#### Neuroimmunoendocrine

The immune system and inflammatory pathways play a significant role in the pathobiology of depression. One simplistic example is interferon-α, which is used to treat medical illness; interferon-α can induce depressive symptoms, and these symptoms are response to pharmacotherapy (reviewed in [123]). Interestingly, studies that have measured cytokines during rTMS response found reversal of the inflammation after rTMS, and one sham-controlled study reported a significant drop in proinflammatory cytokines in the active rTMS group [124••].

To summarize, multiple candidate biomarkers may inform TMS treatment, if replicated. However, for these approaches to be incorporated clinically, they will need to demonstrate reliability, cost-effectiveness, and a meaningful change in clinical response likelihood. As a result, more clinically usable tools need to be developed, and their performance will need to be evaluated in a naturalistic and real-world clinical settings.

## Special section: rTMS during COVID-19 pandemic

The COVID-19 pandemic has inflicted grave consequences on the world, requiring drastic adaptation of clinical care and research, and neuromodulation guidelines have adapted to meet new safety requirements. In addition to adopting best practices (e.g., Center for Disease Control and Prevention recommendations including social distancing, protective personal equipment), special attention can be given to the type of TMS modality applied. For example, various clinics have shifted from standard rTMS to iTBS. As noted above, iTBS has the benefit of being delivered in a few minutes, which provides significantly less exposure between patients and staff, as well as permitting extra time for cleaning. Recently, a group of experts proposed a set of good practices and recommendations for NIBS in the course of the COVID-19 pandemic and for possible future widespread disease outbreaks. Please see reference [125] for further discussion. The pandemic has also reminded the field that the current model of brain stimulation requires regular visits to healthcare settings. This limitation hinders the impact of these interventions, and poses additional risk during pandemic. It is our hope that the current situation prompts the field to revisit the possibility of technologies that permit home use or minimize regular healthcare visits.

## Conclusions

In closing, this review of rTMS for TRD confirms the effectiveness of this technique in improving depressive symptoms, with potential long-lasting effects. Remission occurs on average in one-third of TRD patients, indicating the real-world impact of rTMS. The neurobiological effects of rTMS can be attributed to the direct stimulation of prefrontal areas, with clinical improvement mediated by transsynaptic mechanisms. Regarding different rTMS protocols, the majority of work done to date applied HF-rTMS over the left DLPFC, followed, in order of frequency, by trials delivering LF-rTMS to the right PFC, and fewer using bilateral stimulation. In the last several years, new approaches have emerged, and include dTMS and iTBS, with evidence of efficacy of both techniques, although it remains unclear if any one approach is superior. Furthermore, novel targeting and application procedures continue to develop, each with significant promise to change clinical care. In the setting of the current COVID-19 pandemic, iTBS has gained increased attention for its time-efficient profile, minimizing the chance of exposure. As a final note, in spite of the robust evidence showing rTMS effectiveness, additional studies are needed in order to further investigate predictors of response, potential biomarkers, and the optimal stimulation parameters for TRD.
